# Assessment of Furan and Its Derivatives Intake with Home Prepared Meals and Characterization of Associated Risk for Polish Infants and Toddlers

**DOI:** 10.3390/foods12193618

**Published:** 2023-09-28

**Authors:** Maria Minorczyk, Katarzyna Czaja, Andrzej Starski, Wojciech Korcz, Monika Liszewska, Radosław Lewiński, Mark Gregory Robson, Jacek Postupolski, Paweł Struciński

**Affiliations:** 1 Department of Toxicology and Health Risk Assessment, National Institute of Public Health NIH—National Research Institute, 00-791 Warsaw, Poland; maria.minorczyk@gmail.com (M.M.); kczaja@pzh.gov.pl (K.C.); wkorcz@pzh.gov.pl (W.K.); mliszewska@pzh.gov.pl (M.L.); rlewinski@pzh.gov.pl (R.L.); 2 Department of Food Safety, National Institute of Public Health NIH—National Research Institute, 00-791 Warsaw, Poland; astarski@pzh.gov.pl (A.S.); jpostupolski@pzh.gov.pl (J.P.); 3 Department of Plant Biology, School of Environmental and Biological Sciences, Rutgers University, New Brunswick, NJ 08901, USA; mark.robson@rutgers.edu

**Keywords:** furan, furan derivatives, home-prepared meals, exposure, health risk characterization, infants, toddlers

## Abstract

Furan and its derivatives are found in various heat-treated foods. Furan is classified as a possible human carcinogen. The European Union authorities recommend collecting data on the occurrence of these compounds, estimating consumer exposure, and taking measures to protect human health based on a scientific risk assessment. The aim of this study was to estimate the exposure of infants and toddlers to furan and its methyl derivatives—2-methylfuran, 3-methylfuran, and ∑2,5-dimethylfuran/2-ethylfuran—present in home-prepared foods and to characterize the associated health risks. The compounds of interest were determined using the HS-GC/MS. The risk was characterized by the calculation of the margin of exposure (MoE). Levels of furan and its derivatives in analyzed samples were in the range of <LOD ÷ 10 µg/kg and <LOD ÷ 80.3 µg/kg, respectively. The MoEs for neoplastic effects in most of the presumed scenarios indicate a risk associated with the intake of analyzed compounds in both age groups (MoE < 10,000; 331 to 6354 for 95th percentile, 3181–39,033 for median). The MoEs for non-neoplastic effects indicate a potential risk associated with the intake of 3-methylfuran and Σ2,5-dimethylfuran/2-ethylfuran for high exposure (95th percentile) only (MoE < 100; 16–47). The obtained results indicate the need for further research in this area.

## 1. Introduction

Food safety is one of the key elements of health policy and public health protection activities in the European Union (EU). It is undertaken by risk management bodies, both at the EU and national level.

Among the compounds that are formed during food processing, furan and its derivatives in various heat-treated foods are of great concern [1,2,3,4,5]. Furan is a heterocyclic organic compound. It can be formed in foods from various, naturally occurring precursors via chemical reactions including the thermal decomposition of carbohydrates, thermal oxidation of polyunsaturated fatty acids (PUFA), thermal decomposition of ascorbic acid and its derivatives, and the Maillard reaction involving amino acids and reducing sugars [1,2,6,7,8]. Furan is classified as a possible carcinogenic and cytotoxic substance—category 2B (possible human carcinogen)—by the International Agency for Research on Cancer (IARC) [9]. This fact has resulted in increased efforts to analyze the levels of this contaminant in various food products.

Dietary intake is the main route of exposure to furan and its derivatives. These compounds can also enter the human body via other routes, including inhalation (e.g., during food preparation) and dermal absorption [10,11,12,13,14]. According to the European Food Safety Authority (EFSA), 2-methylfuran and 3-methylfuran contribute significantly to the total dietary intake of furan and its derivatives [2].

Toxicological studies in animals indicate that furan is highly hepatotoxic and moderately nephrotoxic [2,15]. It has a short half-life and is quickly metabolized by cytochrome P-450 enzymes into *cis*-2-butene-1,4-dial (BDA). It has been identified as the key reactive metabolite of furan responsible for its cytotoxic and carcinogenic effects [11,16,17,18,19]. Few pilot animal studies have shown that both methyl derivatives of furan (2-methylfuran and 3-methylfuran) have a similar mechanism of toxic action to the parent substance [2,20].

Estimating the exposure of infants and small children to these compounds is an issue of great importance, as they have a lower metabolic capacity to detoxify contaminants as compared with adults [21,22]. The energy requirements of infants and small children per kilogram of body weight are significantly higher than those of adults [23]. This subpopulation is, therefore, at higher risk associated with exposure to toxic substances for this population. For this reason, many studies have focused on the analysis of furan in foods intended for infants and toddlers [22,24,25,26,27,28,29]. The highest exposure to furan, primarily from ready-made meals, has been estimated for infants [2].

The European Commission and EFSA recommend collecting data on the occurrence of these compounds, estimating consumer exposure, and taking measures to protect human health based on the results of scientific risk assessments [2,30]. It is worth noting that there are very few reports on levels of furan and its derivatives in self-prepared foods [31,32].

The aim of this study was to estimate exposure to furan (F) and its methyl derivatives: 2-methylfuran (2-MF), 3-methylfuran (3-MF), 2,5-dimethylfuran (2,5-DMF), and 2-ethylfuran (2-EF) present in heat-treated baby food prepared at home and characterizing the associated potential health risks for Polish infants and toddlers.

## 2. Materials and Methods

Overview of the study design:

− Meal samples were collected from volunteer parents/guardians; gas chromatography coupled with mass spectrometry (GC/MS) along with a headspace technique (HS) was used for determination of analyzed compounds;
− Risk associated with exposure to chosen contaminants was characterized by margins of exposure (MoE) approach.

### 2.1. Study Material

The study material consisted of 43 samples of home-prepared meals provided by parents/guardians of children aged 6 months to 3 years. The meals were part of their normal diet. The samples came from 31 households. Each sample was accompanied by information about the ingredients used to prepare the meal and how it was prepared. In some cases, one person provided more than one sample. Recruitment of volunteers willing to participate in the study was based on an announcement for National Institute of Public Health NIH—National Research Institute employees.

### 2.2. Description of Analytical Method

Quantitative determination of furan and its derivatives in food requires special caution due to the high volatility of these analytes. The most common technique for determining such substances in food is gas chromatography coupled with mass spectrometry (GC/MS) where the headspace (HS) phase is examined. In our study, the content of furan and its derivatives in the meal samples was determined using the above technique based on the U.S. Food and Drug Administration method [33]. Equipment used included Varian CP3800 gas chromatograph (Varian, Inc., Walnut Creek, CA, USA) with sample injection system for headspace (CTC CombiPal, CTC Analytics AG, Zwingen, Switzerland) coupled to a Varian 4000 MS ion trap mass spectrometer (Varian, Inc., Walnut Creek, CA, USA). MS Workstation 6.9.1 software was used for data integration.

The analyses of the tested compounds applying the gas chromatograph with mass detector were conducted using electron ionization. The following ions were selected and monitored as characteristic of individual compounds in the MS spectrum:

- *m*/*z* = 68 for furan (F);
- *m*/*z* = 72 for d_4_ furan;
- *m*/*z* = 82 for 2-methylfuran and 3-methylfuran (2-MF, 3-MF);
- *m*/*z* = 85 for d_3_ 2-methylfuran and d_3_-3-methylfuran;
- *m*/*z* = 96 for 2-ethylfuran/2,5-dimethylfuran (2-EF, 2,5-DMF);
- *m*/*z* = 101 for d_5_ 2-ethylfuran.
- The settings of the above equipment are shown below:
- Incubation time of the sample before analysis at 60 °C: 20 min;
- A total of 250 μL of headspace phase was introduced into an injector operating at 230 °C in splitless mode;
- Temperature of syringe: 90 °C;
- The separation was conducted using an HP Plot Q (Varian) capillary column: length of 15 m, an internal diameter of 0.32 mm, film thickness of 5 μm;
- Carrier gas: helium, constant flow: 2 mL/min;
- Oven temperature: 50 °C (1 min), 10 °C/min to 225 °C (11.5 min);
- Transfer line temperature: 250 °C, ion trap temperature: 150 °C.

Determination of selected compounds requires particular care to avoid loss of analytes due to the high volatility and the occurrence of analytes at very low levels.

Prior to the analysis of furan and its derivatives, the sample, laboratory vessels, and distilled water were cooled to 4 °C. A portion of 5 g of homogenized sample was weighed out and mixed with water (1:1 m/m). The entire mixture was transferred to a chromatography vial and capped tightly. A standard solution of a given analyte and a standard solution of its deuterated analog were then added using a syringe. An unlabeled standard as well as a deuterated standard of furan (d_4_) and deuterated derivatives: 2-methylfuran d_3_, 2-ethylfuran d_5_, 3-methylfuran d_3_ were used in the study as an internal standard. A calibration curve was prepared for each analyzed sample set. To compensate for the influence of potential losses of analytes associated with their volatility, two replicates of the unfortified and fortified samples were prepared for each of the tested meals [34]. For each sample, the result was corrected for parallel-tested procedural recovery.

### 2.3. Method Validation

For the validation, a single-ingredient sterilized, commercially available, ready-to-eat vegetable (carrot) dish in a glass jar was selected as the matrix. The validation involved determining Limit of Determination, LOD (as 3 standard deviations (SD)), Limit of Quantification, LOQ (as 6 SD), working range, linearity, precision, and recovery. LOQ (LOD) was determined at 0.67 (0.34) µg/kg for furan, 0.60 (0.30) µg/kg for 2-MF µg/kg, 0.38 (0.19) µg/kg for 3-FM, and 1.88 (0.94) µg/kg for ∑2,5-DMF and 2-EF.

The validation was conducted at three assumed fortification levels: 1 µg/kg, 50 µg/kg, 200 µg/kg for F, 3-MF, ∑2.5-DMF, and 2-EF. 2-MF was fortified at levels 5 µg/kg, 200 µg/kg, and 1000 µg/kg because it is found in food samples at higher levels than the other analytes [35,36,37,38].

The following mixtures of standards were used during the validation:

- MIX1: F, 2-MF, 3-MF, 2-EF;
- MIX2: F, 2-EF, 3-MF, 2.5-DMF.

The following mixture of deuterated standard solutions (deuterated MIX) was used to determine recovery:

- MIX deuterated: F, 2-MF, 3-MF, 2-EF.

The exact concentrations of furan and its derivatives in standard solutions and levels of matrix fortification were determined based on weight.

In the cases of 2,5-DMF and 2-EF, due to the fact that both compounds have the same molecular weight and their fragmentation during the analysis causes the same ions with similar retention times, these compounds were determined as a sum: Σ2,5-DMF and 2-EF.

Details of validation results at different fortification levels are presented in Table 1.

Summarizing the key validation results:

- The method’s working range fell within the range of 1–200 μg/kg;
- Coefficients of determination fell within the range from 0.996 to 0.999;
- Recoveries fell within an acceptance criterion of 60–140% adopted for this parameter;
- LODs and LOQs fell within the ranges 0.19–0.94 μg/kg and 0.38–1.88 μg/kg, respectively.

### 2.4. Risk Characterization

The Margin of Exposure (MoE) approach allows risk assessors to assess potential human health concerns arising from exposure to substances, like furan, for which establishing the Toxicological Reference Values (like ADI, Acceptable Daily Intake or TDI, Tolerable Daily Intake) is inappropriate or unfeasible. MoE is the ratio of the dose derived from toxicological studies at which a small but measurable adverse effect is observed to the estimated exposure to the given substance (for a given population). Therefore, in our study, the potential risk was characterized in accordance with the EFSA harmonized approach for risk assessment of genotoxic and carcinogenic substances [39]. For dietary exposure to furan, the estimated daily intake (EDI) and margin of exposure (MoE) were calculated using the equations shown below [29].

The estimated daily intake (EDI):

EDI = test compound content x estimated daily consumption

The margin of exposure (MoE):

MoE = BMDL_10_*/EDI

(BMDL_10_*—benchmark dose lower confidence limit; the dose that produces a predetermined change in the response rate (e.g., increase of 10%) of chosen adverse effect).

As a basis for the risk characterization, two BMDL_10_ values were used, in line with the EFSA approach; one at 1.31 mg/kg bw/day for neoplastic effects (incidence of hepatocellular adenomas or carcinomas) and the other at 0.064 mg/kg bw/day for non-neoplastic effects (incidence of cholangiofibrosis) [2].

In this approach, the risk characterization was considered acceptable in terms of human health risks in cases where the calculated margin of exposure values are >10,000 for neoplastic effects and >100 for non-neoplastic effects [40,41,42].

The assumptions used in this study can be regarded as the so-called worst-case scenario model, which provides the widest possible margin of safety for the consumer [43].

To estimate the dietary exposure to furan and its derivatives, children were divided into two age groups: 6–11 months old (infants) and 12-36 months old (toddlers). These age classes are in line with EFSA classification [2].

The body weights of the infants and toddlers of both sexes were adopted based on percentile charts for Polish boys and girls (50th percentile) [44]. A conservative scenario (upper bound; UB) was used, in which the corresponding numerical values equal to the LOD and LOQ were assumed for all so-called left-censored data, that is for results < LOD as well as < LOQ [45].

The median of the results was considered for estimation of the average exposure to furan and its derivatives. For calculation of the reasonable high intake exposure, the 95th percentile values were used (P95 scenario).

## 3. Results

The samples of home-prepared meals were divided into dishes containing vegetables and meat, dishes containing vegetables only, and fruit dishes. Table 2 shows the concentration range of the analytes determined, the mean, and the median for individual compounds as divided by type of dish.

For the estimation of exposure of infants and toddlers to furan and its methyl derivatives, the median and 95th percentile concentrations of the tested compounds and the average portion weight taken from the national dietary recommendations were considered [46].

The data used to estimate exposure to furan and its methyl derivatives are presented in Table 3. The exposure was estimated according to the age disaggregation of children for whom all categories of meals were prepared. For estimating the daily intake for the age range 6–11 months, the body weight for an 8-month-old infant (7.9 kg—girl; 8.6 kg—boy) was considered, while for the age range 12–36 months, the body weight for a 20-month-old child (10.6 kg—girl; 11.3 kg—boy) was used.

The calculated values of the estimated daily intake for the assumed scenarios for furan and its methyl derivatives are summarized in Table 4.

The highest estimated dietary exposure to furan and 2-MF (P95) was obtained for girls aged 12–36 months. With regards to 3-MF, the highest exposure value was found in the population of boys over 12 months old. In the case of Σ2,5-DMF and 2-EF, the most exposed group was girls under the age of 12 months.

Table 5 and Table 6 present the calculated margins of exposure for furan and individual derivatives for non-neoplastic and neoplastic effects, respectively.

The calculated margins of exposure for the assumed scenarios for non-neoplastic effects indicate that there may be a health risk associated with the intake of 3-MF with food by infants and toddlers (for the P95 level). Similarly, for Σ2,5-DMF and 2-EF, the margins of exposure calculated for P95 indicate a health risk for boys and girls under one year of age. In the other scenarios assumed for derivatives found in self-prepared meals, the calculated MoEs were >100. For furan, no health risk was identified for non-neoplastic effects.

The margins of exposure for neoplastic effects of less than 10,000 indicate a potential risk. Such results were found in most of the scenarios assumed for all the tested compounds consumed with home-prepared meals. Only for furan and the sum of two derivatives Σ2,5-DMF and 2-EF, the MoE values considering the average intake do not indicate the health risk for infants and toddlers of both sexes.

## 4. Discussion

The results obtained confirm the widespread occurrence of furan and its derivatives in foods intended for infants and toddlers. The results of our study as well as the number of available literature references seem too small to make a comparison between the levels of furan and its derivatives in home-prepared meals and explicitly state that such meals pose a low health risk to this group of consumers. One of the few available scientific publications describing the determination of furan and its derivatives in self-prepared meals for children is authored by Bianchi et al. [31]. The authors determined furan levels in seven types of homemade baby food, which were <LOD or very low (the highest result was 1 µg/kg in cooked peas). In another study, authors showed that, in contrast to commercially available heat-treated preparations, none of the 20 home-prepared meals for children contained furan above the detection limit of the method used [32]. Based on the literature search, no articles presenting results for the determination of furan methyl derivatives in homemade meal samples were found.

Based on the results of our study it can be concluded that the levels of furan and its derivatives found in the samples of self-prepared meals are lower compared to the corresponding levels obtained from analyses of ready-to-eat baby foods [2]. This suggests that the content of furan and its derivatives in samples of home-prepared meals may be influenced by the method of their preparation. Each food preparation method may be characterized by different temperatures and heat treatment duration. Whether dishes were cooked covered or uncovered may also be relevant. Reducing the levels of furan and its derivatives in food can be achieved through measures aimed at reducing their formation or by removing these compounds; however, it should be borne in mind that it is difficult to reduce their formation without affecting the organoleptic properties of the food [47]. Research on reducing furan formation has addressed both food production [48,49] and food preparation for consumption [50,51,52]. Most home cooking methods reduce the content of these compounds. This effect can also be achieved by leaving the dish in an open vessel for a while as well as stirring it [52,53]. As suggested by the authors of those studies, when it comes to ready-made meals, heating and stirring would be a simple method to reduce furan levels in children’s food [54,55]. Studies on this phenomenon have shown furan reductions of 29–55% in vegetable purees during various microwave heating procedures [56], and even reductions of up to 85% recorded when open jars were heated for a relatively longer time in boiling water, as well as reductions of about 50% if the baby food jar was left open but not heated [57]. The lower results obtained in this study in relation to the compounds tested in home-prepared meals compared to the corresponding results for ready-to-eat baby foods may support the conclusions reached in the aforementioned work.

It needs to be emphasized that the analytical procedure and calculations adopted do not consider the fact that a meal is heated and mixed before being served to the child. These parameters are difficult to model because each parent has different habits when preparing a meal for their child. It can therefore be assumed that the risks characterized in our study article are overestimated. Due to the lack of a universal approach for developing exposure scenarios, it is difficult to compare the results of this study with others. The calculation methods adopted and certain assumptions (e.g., when developing exposure scenarios, including age and body weight, diet composition, and portion size) are different in other studies. Different toxicological reference values (ADI, TDI, BMDL) are also used by other authors.

It should also be added that the dietary exposure scenarios for furan do not include additional inhalation exposure that occurs during food preparation and cooking [58]. The results presented are low and indicate that they depend on a number of factors, including the cooking technique used and its duration, as well as the room size.

## 5. Conclusions

The margins of exposure (MOEs) calculated for most scenarios assumed for home-cooked meals indicate a health risk for infants and toddlers associated with exposure to furan and its derivatives. For non-neoplastic effects the risk (MoE < 100) was obtained for 3-MF, Σ2,5-DMF, and 2-EF for both age groups (P95 scenario). MoE values <10,000 for neoplastic effects were found for all scenarios except the median scenario for furan, Σ2,5-DMF, and 2-EF. The estimated risks can be, however, considered as calculated with a large margin of uncertainty due to the conservative approach used in this study.

It is necessary to monitor the exposure of infants and small children to these compounds ingested with food. It is of particular importance to analyze the levels of furan and its derivatives in home-prepared foods for this subpopulation to fill in the existing data gap.

Further studies on the impact of factors such as time and methods of heating food, cooking in covered or uncovered pots, temperature, stirring the meal during cooking, and storage conditions of ingredients before cooking on the level of furan and its derivatives in food prepared for children at home are necessary. The results of such studies would enable the identification of risk-mitigation measures that could be implemented by parents/guardians.

To ensure consistency of results, it is advisable that universal exposure scenarios be developed, and risks associated with the dietary intake of contaminants by infants and small children be characterized.

## Figures and Tables

**Table 1:** Summary of validation results at different fortification levels (N = 10).

F	Mean [µg/kg]	SD	RSD [%]	LOD	LOQ	Recovery [%]
				[µg/kg]		
Matrix (not fortified)	0.66	0.11	17.16	0.34	0.67	—
Fortification level 1µg/kg MIX1	2.40	0.30	12.42			112.74
Fortification level MIX2	2.52	0.28	11.09			117.68
Fortification level 50 µg/kg MIX1	44.61	2.16	4.84			87.41
Fortification level 50 µg/kg MIX2	49.69	2.88	5.79			97.79
Fortification level 200 µg/kg MIX1	203.63	11.27	5.53			94.73
Fortification level 200 µg/kg MIX2	199.69	13.84	6.93			93.88
**2-MF**	**Mean**	**SD**	**RSD**	**LOD**	**LOQ**	**Recovery** **[%]**
Matrix (not fortified)	0.99	0.10	10.09	0.30	0.60	—
Fortification level 1µg/kg MIX1	6.88	1.30	18.93			81.81
Fortification level MIX2	10.15	0.78	7.66			124.35
Fortification level 50 µg/kg MIX1	158.22	12.86	8.12			67.20
Fortification level 50 µg/kg MIX2	180.35	12.52	6.94			76.91
Fortification level 200 µg/kg MIX1	999.37	95.48	9.55			100.14
Fortification level 200 µg/kg MIX2	957.51	73.45	7.67			97.01
**3-MF**	**Mean**	**SD**	**RSD**	**LOD**	**LOQ**	**Recovery** **[%]**
Matrix (not fortified)	0.29	0.06	22.06	0.19	0.38	—
Fortification level 1µg/kg MIX1	1.33	0.26	19.72			65.36
Fortification level MIX2	1.55	0.13	8.28			75.03
Fortification level 50 µg/kg MIX1	44.69	3.31	7.41			85.46
Fortification level 50 µg/kg MIX2	52.55	3.18	6.04			98.35
Fortification level 200 µg/kg MIX1	222.13	18.83	8.47			100.19
Fortification level 200 µg/kg MIX2	222.14	18.89	8.50			98.73
**∑2.5-DMF and 2-EF**	**Mean**	**SD**	**RSD**	**LOD**	**LOQ**	**Recovery** **[%]**
Matrix (not fortified)	<LOD	—	—	0.94	1.88	—
Fortification level 1µg/kg MIX1	1.65	0.31	19.05			—
Fortification level MIX2	1.52	0.23	15.03			103.22
Fortification level 50 µg/kg MIX1	42.75	2.88	6.73			85.72
Fortification level 50 µg/kg MIX2	46.25	3.9	8.46			99.13
Fortification level 200 µg/kg MIX1	213.17	20.33	9.54			100.30
Fortification level 200 µg/kg MIX2	190.87	17.98	9.42			96.76

**Table 2:** The levels of furan and its derivatives in home-prepared meals.

Analyte Tested	Range [µg/kg]	Mean [µg/kg]	Median [µg/kg]
Vegetables and meat (n = 21)			
F	0.9–4.3	2.4	1.9
2-MF	1.4–25.9	5.6	4.7
3-MF	1.8–28.2	4.6	4.3
Σ2,5-DMF/2-EF	1.4–72.0	10.3	1.6
Vegetables (n = 16)			
F	2.7–4.5	3.7	3.8
2-MF	0.6–14.7	7.1	7.3
3-MF	1.6–74.6	18.2	7.5
Σ2,5-DMF/2-EF	4.4–80.3	31.1	8.6
Fruit (n = 3)			
F	0.9–2.0	1.5	–
2-MF	7.4–10.2	8.8	–
3-MF	3.3–5.8	4.4	3.9
Σ2,5-DMF/2-EF	<LOD	–	–
Other (n = 3)			
F	10	–	–
2-MF	7.3	–	–
3-MF	2.4–31.9	13.1	4.9
Σ2,5-DMF/2-EF	2.4	–	–

**Table 3:** Levels of furan and its derivatives (median and 95th percentile) in analyzed samples taking into account the age for which the dish was prepared.

Derivative	Age Range	Median Result [µg/kg]	P95 Result [µg/kg]
F	6–11 months	0.7	4.3
	12–36 months	0.7	4.2
2-MF	6–11 months	4.5	11.5
	12–36 months	7.0	9.5
3-MF	6–11 months	4.7	28.4
	12–36 months	4.7	31.4
Σ2.5-DMF and 2-EF	6–11 months	0.9	72.0
	12–36 months	0.9	4.8

**Table 4:** The estimated daily intake of furan and its derivatives [µg/kg bw/day]. Bold data reflects the highest estimated exposure to analyzed compounds.

UB (Upper Bound)	Girls	Boys	Girls	Boys
**Median scenario**	F		2-MF	
6–11 months	0.0336	0.0310	0.2205	0.2038
12–36 months	0.0412	0.0385	0.4118	0.3853
**P95 scenario**				
6–11 months	0.2062	0.1905	0.5514	0.5095
12–36 months	**0.2471**	0.2312	**0.5588**	0.5229
**Median scenario**	3-MF		Σ2.5-DMF and 2-EF	
6–11 months	0.2253	0.2082	0.0432	0.0399
12–36 months	0.2765	0.2587	0.0529	0.0495
**P95 scenario**				
6–11 months	1.3616	1.3911	**3.4521**	3.1899
12–36 months	1.8471	**3.9633**	0.2824	0.2642

**Table 5:** Margins of exposure for non-neoplastic effects based on the adopted scenarios. Bold data reflects the MoE values that indicate risk (<100).

BMDL_10_ = 0.064 mg/kg bw/Day	MoE Girls	MoE Boys	MoE Girls	MoE Boys	MoE Girls	MoE Boys	MoE Girls	MoE Boys
**Median scenario**	F		2-MF		3-MF		Σ2.5-DMF and 2-EF	
6–11 months	1907	2064	290	314	284	307	1483	1605
12–36 months	1554	1661	155	166	231	247	1209	1292
**P95 scenario**	F		2-MF		3-MF		Σ2.5-DMF and 2-EF	
6–11 months	310	336	116	126	**47**	**46**	**19**	**20**
12–36 months	259	277	115	122	**35**	**16**	227	242

**Table 6:** Margins of exposure for neoplastic effects based on the adopted scenarios. Bold data reflects the MoE values that indicate risk (<10,000).

BMDL_10_ = 1.31 mg/kg bw/Day	MoE Girls	MoE Boys	MoE Girls	MoE Boys	MoE Girls	MoE Boys	MoE Girls	MoE Boys
**Median scenario**	F		2-MF		3-MF		Σ2.5-DMF and 2-EF	
6–11 months	39,033	42,241	**5940**	**6428**	**5813**	**6291**	30,359	32,854
12–36 months	31,814	33,998	**3181**	**3400**	**4738**	**5063**	24,744	26,443
**P95 scenario**	F		2-MF		3-MF		Σ2.5-DMF and 2-EF	
6–11 months	**6354**	**6876**	**2376**	**2571**	**962**	**942**	**379**	**411**
12–36 months	**5302**	**5666**	**2344**	**2505**	**709**	**331**	**4640**	**4958**

## Data Availability

Data is contained within the article.
